# Functional and Oncological Outcomes Following Robot-Assisted and Laparoscopic Radical Prostatectomy for Localized Prostate Cancer With a Large Prostate Volume: A Retrospective Analysis With Minimum 2-Year Follow-Ups

**DOI:** 10.3389/fonc.2021.714680

**Published:** 2021-09-23

**Authors:** Wen Deng, Xiaoqiang Liu, Weipeng Liu, Cheng Zhang, Xiaochen Zhou, Luyao Chen, Ju Guo, Gongxian Wang, Bin Fu

**Affiliations:** ^1^ Department of Urology, The First Affiliated Hospital of Nanchang University, Nanchang, China; ^2^ Jiangxi Institute of Urology, Nanchang, China

**Keywords:** radical prostatectomy, prostate cancer, large prostate, robot, laparoscopic

## Abstract

**Objective:**

We aimed to analyze the perioperative, functional, and oncologic outcomes following robot-assisted radical prostatectomy (RARP) and laparoscopic radical prostatectomy (LRP) for patients with localized prostate cancer (PCa) characterized by a large prostate volume (PV; ≥50 ml) over a minimum of 2 years follow-up.

**Materials and Methods:**

Patients undergoing RARP and LRP for localized PCa with a large PV were included in the final analysis. The perioperative, functional, and oncologic outcomes were analyzed between the two groups.

**Results:**

All operations were successfully completed without open conversion in both groups. The mean operative time and estimated blood loss in the RARP group were significantly decreased compared to those in the LRP group (139.4 *vs*. 159.0 min, *p* = 0.001, and 124.2 *vs*. 157.3 ml, *p* = 0.003, respectively). Patients in the RARP arm had significantly lower proportions of grade II or lower and of higher than grade II postoperative complications compared with those in the LRP group (7.9% *vs*. 17.1%, *p* = 0.033, and 1.6% *vs*. 6.7%, *p* = 0.047, respectively). No significant differences in terms of the rates of pT3 disease, positive surgical margin, and positive lymph node were noted between the two groups. Moreover, no significant difference in the median specimen Gleason score was observed between the RARP and LRP groups (6 *vs*. 7, *p* = 0.984). RARP *vs*. LRP resulted in higher proportions of urinary continence upon catheter removal (48.4% *vs*. 33.3%, *p* = 0.021) and at 3 (65.1% *vs*. 50.5%, *p* = 0.025) and 24 (90.5% *vs*. 81.0%, *p* = 0.037) months post-operation. The median erectile function scores at 6 and 24 months post-operation in the RARP arm were also significantly higher than those in the LRP arm (15 *vs*. 15, *p* = 0.042, and 15 *vs*. 13, *p* = 0.026, respectively). Kaplan–Meier analyses indicated that the biochemical recurrence-free survival and accumulative proportion of continence were statistically comparable between the two groups (*p* = 0.315 and *p* = 0.020, respectively).

**Conclusions:**

For surgically managing localized PCa with a large prostate (≥50 ml), RARP had a tendency toward a lower risk of postoperative complications and better functional preservation without cancer control being compromised when compared to LRP.

## Introduction

Prostate cancer (PCa), accounting for 15% of all cancers (1), represents one of the most prevalent cancer entities and the fifth leading cause of cancer-specific death among men (2). The rate of patients diagnosed with localized PCa has dramatically increased following the extensive implementation of prostate-specific antigen (PSA) screening (3). Prostate enlargement, a very common condition among the aging male population, has demonstrated increased prevalence over the years (4, 5). The mean size of prostates removed during radical prostatectomy (RP) has proportionately increased compared to that before the widespread application of PSA testing (6). Prostate volume (PV) is considered a predictor of adverse disease features and disease recurrence after RP (7). Larger PVs are closely associated with limited mobility in cases of small pelvis and narrowed visualization during RP, consequently posing considerable challenges to treatment targeting functional protection and oncologic control (8, 9).

RP, a curative treatment for organ-confined PCa, aims to radically remove localized PCa while, whenever possible, retaining urinary continence (UC) and erectile function (EF) (10). The technical development of RP involved laparoscopic RP (LRP) and robot-assisted RP (RARP). LRP has rapidly emerged as an alternative to open RP, with the advantage of reducing blood loss and length of hospital stay (1, 11). Subsequently, with the superiority of robotic surgical platforms in providing a three-dimensional magnified visualization of the surgical field, improved dexterity, and high precision, RARP is generally considered an excellent evolution of minimally invasive surgery to address the difficulties inherent in complex laparoscopic surgery (12) and has been widely adopted for localized PCa since 2001 (13–15). However, given the prohibitively high cost of robotic systems and the scarcity of scientific evidence supporting the benefits of RARP over LRP, LRP is still routinely performed for localized PCa in many centers across Europe and Asia (16, 17). Furthermore, the controversy on whether the superiority of RARP mentioned above can mitigate the surgical challenges of LRP and contribute to superior functional protection and cancer control for PCa patients remains due to the lack of high-level relevant evidence. Thus far, only three randomized controlled trials (RCTs) (18–20) have compared RARP and LRP for localized PCa, with different endpoints; however, these trials featured short-term study periods and reported conflicting results, which is far from reaching a convincing consensus on this topic. With regard to PCa patients with large PVs, it has never been investigated whether the high expectations of RARP over LRP were warranted, even though the issue is of clinical importance.

The definition of large prostate varies widely among different published studies. It has been indicated that a PV of >50 ml might be taken into consideration for the biopsy decision-making in the Chinese population with total PSA (tPSA) ranging from 4 to 20 ng/ml (21). In addition, many studies regard 50 ml as a cutoff value to define large PV when assessing the impacts of PV on surgical and oncological outcomes following RP (22, 23). Considering the clinical significance of PV ≥ 50 ml in the detection and treatment of PCa, the same PV was considered as the cutoff value for defining a large prostate in the present study.

To occlude the wide gap of scientific evidence regarding the functional and oncological outcomes following RARP and LRP for localized PCa with a large PV, we designed this first analysis documenting the differences in the perioperative, functional, and oncologic outcomes obtained after RARP and LRP for localized PCa with a large PV (≥50 ml) with at least 2 years of follow-up in a retrospective fashion.

## Materials and Methods

### Data Source and Patient Selection

All the demographic, clinical, and pathologic information of patients undergoing RARP or LRP for eradicating localized PCa between March 2015 and March 2019 were retrospectively collected from our prospectively maintained database with the approval of the Institutional Review Board and Ethics Committee of the First Affiliated Hospital of Nanchang University. Patients with PCa were enrolled into this study when they met the following inclusion criteria: 1) receiving RARP or LRP for localized PCa; 2) PV ≥50 ml calculated by transrectal ultrasound; and 3) absence of any clinical evidence of positive lymph nodes or T3–T4 stage. Only when all of these eligibility criteria were simultaneously satisfied was the instance included in the final comparison; patients failing to satisfy at least one of these criteria were excluded from the study. All cases were routinely evaluated preoperatively by prostate magnetic resonance imaging, bone scintigraphy, and abdominal computed tomography.

### Technical Considerations

Both RARP and LRP were carried out *via* the anterior method by two highly experienced surgeons (FB and WG), both of whom had completed more than 400 LRPs and 200 RARPs as an operator or a trainee prior to the initiation of the study periods. All patients were fully informed of the indications and procedures of RARP and LRP, the differences between these techniques, alternative choices for cancer management, and the costs of different treatments and were then provided the written informed consent including all the information mentioned above. Eventually, the surgeons generally recommended the most appropriate approach on the basis of the features of the tumors, such as PV and risk stratification, and the patients’ conditions, such as economic capacity. All surgeries were conducted after the acquisition of written informed consent from each patient in both arms.

The modified technique established by Menon et al. (24) was followed to conduct the anterior approach to RARP, while the surgical steps described by Touijer et al. (25) were applied to perform the anterior approach to LRP. Posterior reconstruction was routinely done in all cases in both arms. Patients with a preoperative estimated risk exceeding 5% in lymph node invasion routinely received an anatomically extended pelvic lymph node dissection (ePLND); ePLND is usually omitted for those with a lower risk of nodal involvement according to the surgeons’ clinical judgment. A standardized extended PLND template, including removal of the nodes overlying the external iliac artery and vein, the nodes within the obturator fossa, the nodes medial and lateral to the internal iliac artery, and the nodes overlying the common iliac artery and vein up to the ureteral crossing, was utilized in all cases receiving lymph node dissections. Application of the nerve-sparing technique was preoperatively arranged on the grounds of clinical features and intraoperatively altered depending on the evidence of bundle invasion.

### Variable Definition and Endpoints

All information regarding the preoperative demographics, such as age, body mass index (BMI), diabetes mellitus, hypertension, American Society of Anesthesiologists score, preoperative tPSA, and preoperative EF evaluated with the International Index of Erectile Function (IIEF)-5 score (26), and the clinical disease variables, such as clinical TNM stage, biopsy Gleason score, and PV evaluated by transrectal ultrasound, were gathered from the database.

Details of the perioperative outcomes, such as the operative time (OT), estimated blood loss (EBL), ePLND, nerve-sparing technique, open conversion, transfusion, postoperative hospital stay, and postoperative complications graded according to the Clavien–Dindo classification (27), and the pathologic outcomes, such as pathologic T stage, specimen Gleason score, positive surgical margin (PSM), and positive lymph nodes, were also retrieved from our database. Other means, such as chart reviews, outpatient visits, and telephone interviews, were employed to obtain information on postoperative complications, as necessary.

Postoperative follow-up was regularly conducted every 3 months within the first year after surgery and then every 6 months in the second year onward. Postoperative PSA tests were routinely conducted every 3 months for each patient to detect biochemical recurrence (BCR), which was considered on condition that two consecutive rising serum PSA values were 0.2 ng/ml or greater in two separate detections. UC was regarded as the prophylactic use of one dry pad or the absence of any pad within a day. The tPSA level and the EF scores were presented at 12 and 24 months post-operation, while the proportion of UC recovery was compared upon catheter removal and at 3, 12, and 24 months post-operation. For each patient receiving RP, the IIEF-5 score questionnaire was routinely completed before surgery and at each postoperative follow-up visit. Full EF recovery was defined as IIEF-5 score ≥17 over 12 months after surgery (28).

### Statistical Analysis

All normally distributed continuous variables were presented as mean and standard deviation and compared with the application of independent *t*-tests. Other continuous variables were expressed as median and interquartile range (IQR) and analyzed by the Wilcoxon rank-sum test. All categorical variables were recorded as proportion and percentage and analyzed using Pearson’s chi-square test or Fisher’s exact test. Estimated BCR-free survival probabilities and proportions of UC recovery were compared *via* the Kaplan–Meier method. STATA version 12.0 (STATA Corp., College Station, TX, USA) was employed for all statistical analyses, with a two-sided *p*-value <0.05 denoting statistical significance.

## Results

Over the study period, enrolled in the final analysis in accordance with the inclusion criteria were a total of 231 eligible and consenting patients, of whom 126 and 105 were classified by surgical approaches into the RARP arm and the LRP arm, respectively. All preoperative variables regarding the clinical and tumor features are summarized in **Table 1**. No statistically significant differences were observed between the two arms in terms of age at surgery, BMI, tPSA, PV, proportions of diabetes mellitus and hypertension, distribution of clinical T stage, preoperative IIEF-5 score, and biopsy Gleason score.

**Table 1:** Preoperative characteristics by surgery type.

Variable	RARP (*n* = 126)	LRP (*n* = 105)	*p*-value
Age (years), mean (SD)	66.6 (7.9)	65.2 (5.5)	0.204
BMI (kg/m^2^), mean (SD)	22.3 (3.8)	23.1 (3.5)	0.145
Diabetes mellitus (yes), *n* (%)	15 (11.9%)	15 (14.3%)	0.592
Hypertension (yes), *n* (%)	30 (23.8%)	29 (27.6%)	0.509
Preoperative tPSA (ng/ml), mean (SD)	19.4 (10.1)	18.7 (7.6)	0.598
Prostate volume (ml), mean (SD)	69.6 (13.8)	66.4 (13.6)	0.138
Preoperative IIEF-5 score, median (IQR)	18 (16–21)	18 (15–21)	0.310
cTNM stage, *n* (%)			0.182
T1	59 (46.8%)	58 (55.2%)	
T2a–b	45 (35.7%)	37 (35.2%)	
T2c	22 (17.5%)	10 (9.6%)	
Biopsy Gleason score, median (IQR)	6 (5–8)	7 (6–7)	0.509

SD, standard deviation; BMI, body mass index; tPSA, total prostate-specific antigen; IIEF, International Index of Erectile Function; cTNM, clinical TNM; IQR, interquartile range.

The perioperative results and pathologic features are delineated in **Table 2**. All operations were successfully completed without open conversion in both groups. The mean OT and EBL in the RARP group were significantly decreased compared to those in the LRP group (*p* = 0.001 and *p* = 0.003, respectively). ePLND was conducted in 24 (19.0%) cases in the RARP group and in 16 (15.2%) cases in the LRP group (*p* = 0.446), while lymph node invasion was detected in 14 (11.1%) and 9 (8.6%) cases in the RARP and LRP groups, respectively (*p* = 0.521). The median (IQR) values of lymph nodes removed from patients in the RARP and LRP groups were 8 (0–14) and 7 (0–13), respectively (*p* = 0.717). The nerve-sparing technique was done in 86 (68.3%) patients in the RARP group and in 65 (61.9%) patients in the LRP group (*p* = 0.313). Patients in the LRP group had significantly higher rates of transfusion than those in the RARP group (8.6% *vs*. 2.4%, *p* = 0.035). No significant differences in the distribution of pathologic T stage, PSM rate, and median specimen Gleason score were noted between the RARP and LRP groups (*p* = 0.199, *p* = 0.248, and *p* = 0.984, respectively). Among patients with pT2 disease, the PSM rate was 10.8% in the RARP group and was 17.6% in the LRP group (*p* = 0.186). Among patients with pT3 disease, PSM was detected in 8 (24.2%) and 6 (30.0%) patients in the RARP and LRP groups, respectively (*p* = 0.645). Patients undergoing LRP tended toward a higher risk of grade II or lower and of higher than grade II postoperative complications compared with those receiving RARP (*p* = 0.033 and *p* = 0.047, respectively). However, no significant difference in the median length of hospital stay was found between the LRP and RARP groups (*p* = 0.537). Notably, the mean hospital cost in the RARP group was significantly higher than that in the LRP group (US $6950 *vs*. US $4533, *p* < 0.001).

**Table 2:** Perioperative outcomes following robot-assisted radical prostatectomy (RARP) and laparoscopic radical prostatectomy (LRP).

Variable	RARP (*n* = 126)	LRP (*n* = 105)	*p*-value
Operative time (min), mean (SD)	139.4 (25.4)	159.0 (31.3)	0.001
Estimated blood loss (ml), mean (SD)	124.2 (71.6)	157.3 (66.4)	0.003
ePLND, *n* (%)	24 (19.0%)	16 (15.2%)	0.446
Nerve sparing technique, *n* (%)	86 (68.3%)	65 (61.9%)	0.313
Open conversion, *n* (%)	0 (0%)	0 (0%)	–
Transfusion, *n* (%)	3 (2.4%)	9 (8.6%)	0.035
Postoperative pathology			
Pathological T stage			0.199
pT2, *n* (%)	93 (73.8%)	85 (81.0%)	
pT3, *n* (%)	33 (26.2%)	20 (19.0%)	
Specimen Gleason score, median (IQR)	6 (5, 8)	7 (5, 7)	0.984
Overall positive surgical margin, *N* (%)	18 (14.3%)	21 (20.0%)	0.248
pT2 disease, *n* (%)	10 (10.8%)	15 (17.6%)	0.186
pT3 disease, *n* (%)	8 (24.2%)	6 (30.0%)	0.645
Total number of removed lymph nodes, median (IQR)	8 (0–14)	7 (0–13)	0.717
Positive lymph node, *n* (%)	14 (11.1%)	9 (8.6%)	0.521
Postoperative complications, *N* (%)	12 (9.5%)	25 (23.8%)	0.003
Grade II or lower, *n* (%)	10 (7.9%)	18 (17.1%)	0.033
Higher than grade II, *n* (%)	2 (1.6%)	7 (6.7%)	0.047
Length of hospital stay (days), median (IQR)	14 (14–15)	15 (13–15)	0.537
Hospitalization cost (USD), mean (SD)	6,950 (655)	4,533 (827)	<0.001

ePLND, extended pelvic lymph nodes dissection; SD, standard deviation; IQR, interquartile range.

**Figure 1:**
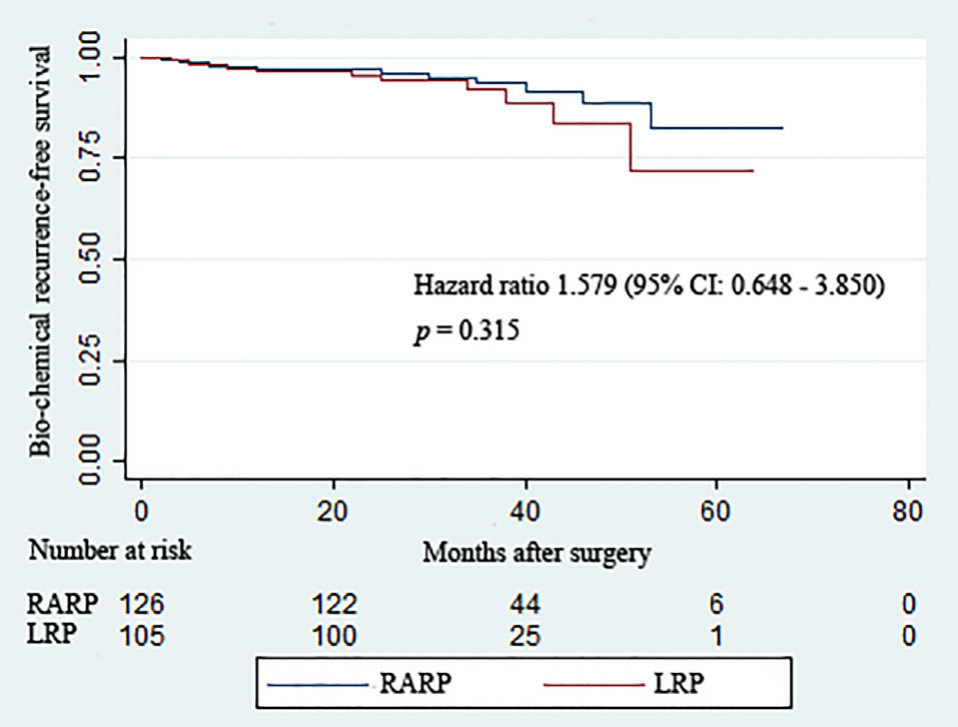
Kaplan–Meier curves showing the biochemical recurrence-free survival rates following robot-assisted and laparoscopic radical prostatectomy for prostate cancer with a large prostate volume over the follow-up duration.

All patients included in this analysis were followed up for at least 2 years after surgery. The median follow-up durations of the RARP and LRP arms were 36.8 and 32.8 months, respectively. Statistical comparability was also noticed with respect to the mean serum PSA at 12 and 24 months post-operation (*p* = 0.951 and *p* = 0.795, respectively) (**Table 3**). Ten patients in the RARP group and nine patients in the LRP group experienced BCR within the follow-up period. The Kaplan–Meier curve shown in **Figure 1** reveals no significant difference in the BCR-free survival rates following RARP and LRP for localized PCa with a large PV (*p* = 0.315).

**Table 3:** Postoperative outcomes following robot-assisted radical prostatectomy (RARP) and laparoscopic radical prostatectomy (LRP).

Variable	RARP (*n* = 126)	LRP (*n* = 105)	*p*-value
Oncology: postoperative tPSA (ng/ml)			
Postoperative 12 months, mean (SD)	0.194 (0.901)	0.202 (0.827)	0.951
Last follow-up, mean (SD)	0.717 (2.305)	0.719 (2.298)	0.795
Urinary continence			
Continent on removal of catheter, *n* (%)	61 (48.4%)	35 (33.3%)	0.021
Continent at postoperative 3 months, *n* (%)	82 (65.1%)	53 (50.5%)	0.025
Continent at postoperative 12 months, *n* (%)	114 (90.5%)	85 (81.0%)	0.037
Continent at postoperative 24 months, *n* (%)	114 (90.5%)	85 (81.0%)	0.037
Erectile function			
IIEF-5 score at postoperative 6 months, median (IQR)	15 (10–18)	15 (9–17)	0.042
IIEF-5 score at postoperative 12 months, median (IQR)	16 (10–19)	14 (9–18)	0.031
IIEF-5 score at postoperative 24 months, median (IQR)	15 (9–18)	13 (9–16)	0.026
Full potency recovery at postoperative 24 months, *n* (%)	53 (42.1%)	30 (28.6%)	0.033

tPSA, total prostate-specific antigen; SD, standard deviation; IIEF, International Index of Erectile Function; IQR, interquartile range.

**Figure 2:**
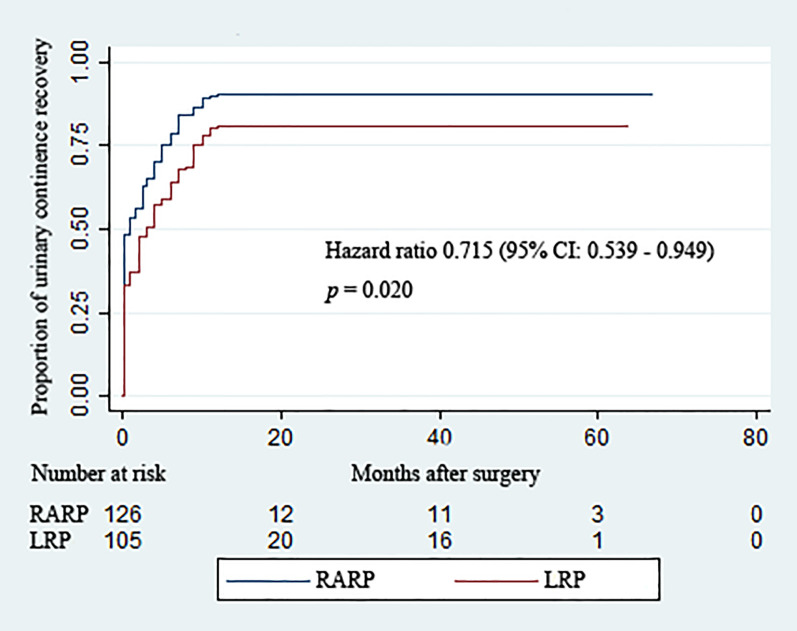
Kaplan–Meier curves showing the proportions of urinary continence (UC) following robot-assisted and laparoscopic radical prostatectomy for prostate cancer with a large prostate volume over the follow-up duration. UC was defined as requiring no pad or preventively using one dry pad per day.

Removal of Foley catheters was routinely carried out at postoperative 2 weeks in both groups. **Table 3** summarizes the continence rates of the two groups at different time points. The proportion of patients achieving continence in the RARP group was significantly higher than that in the LRP group upon catheter removal (48.4% *vs*. 33.3%, *p* = 0.021) and at 3 (65.1% *vs*. 50.5%, *p* = 0.025), 12 (90.5% *vs*. 81.0%, *p* = 0.037), and 24 (90.5% *vs*. 81.0%, *p* = 0.037) months post-operation. Over the complete duration of the follow-up period, the accumulative likelihood of UC recovery was significantly higher in the RARP arm than in the LRP group (*p* = 0.020) (**Figure 2**).

As revealed in **Table 3**, statistically significant differences were found between the RARP and LRP groups in terms of the median IIEF-5 score at 6 (15 *vs*. 15, *p* = 0.042), 12 (16 *vs*. 14, *p* = 0.031), and 24 (15 *vs*. 13, *p* = 0.026) months post-operation, and a total of 53 (42.1%) patients in the RARP group and 30 (28.6%) patients in the LRP group achieved potency recovery at 24 months post-operation (*p* = 0.033), demonstrating the superiority of RARP over LRP in preserving EF for patients with localized PCa with a large PV.

## Discussion

Prostate enlargement is increasingly prevalent in the aging male population (4). A large PV poses enormous challenges in efforts to achieve favorable outcomes in functional preservation and oncological control during RP. More recently, LRP and RARP had been developed. However, whether the advantages of RARP over LRP can bring about better functional preservation and cancer control for PCa patients with a large PV has not been discussed to date, which is of clinical importance. This study is the first to compare the effects of RARP and LRP for localized PCa with a large PV. Our results collectively demonstrate the superiority of RARP over LRP in promoting UC recovery and preserving EF without compromising cancer cure for localized PCa with a large PV (≥50 ml).

The significant reduction in the mean OT of RARP *vs*. LRP may be mainly attributed to the advantage of RARP over LRP in achieving faster anastomosis during RP (29), especially for PCa with a large PV. Actually, the improved dexterity and high precision of the robotic platform may also assist in the removal of large prostates within a limited surgical field. Significant differences in the mean EBL and transfusion rate may be explained by the enhanced visualization and dexterity afforded by robotic surgery, which could help minimize bleeding in a timely fashion. In spite of the higher rates of ePLND and the nerve-sparing technique applied in the RARP group compared to that in the LRP group, the moderate differences of these factors failed to acutely increase the mean OT and EBL required for RARP. It was not strange that the slightly more applications of ePLND and the nerve-sparing technique by means of robotic platform did not significantly impact the perioperative outcomes pertaining to the mean OT and EBL of the entire cohort in the RARP group in highly experienced hands. The impact of surgeons’ experience, another important factor influencing perioperative outcomes, was extremely limited between the two groups in this analysis due to the similar levels of expertise of the two surgeons performing all surgeries.

Regarding the safety outcomes, the risks of grade II or lower and of higher than grade II postoperative complications in the LRP group were significantly higher than those in the RARP group, which may be explained by the lower invasiveness and risk of organ injury with RARP (29). In the present study, 25 (23.8%) patients undergoing LRP developed complications of any grade, while the percentages of postoperative complications following LRP for localized PCa ranged from 3.9% to 21.8% in published studies (29), coordinating the cautions that more invasive operations have greater risks of adverse events. Intriguingly, the possibility of postoperative complications in the RARP group was significantly lower than that in the LRP group, even though both ePLND and the nerve-sparing technique were more frequently completed in patients undergoing RARP, implying the benefits of robotics in reducing the incidence of adverse events compared with LRP for patients with PCa with a large prostate.

Surgical treatment for cancer should be tempered with a critical analysis of the expected oncologic outcomes. Our results revealed a trend toward higher PSM rates after LRP (20.0%) compared with RARP (14.3%) among PCa patients with a large prostate. However, the trend did not achieve a significant difference between the two groups. Similar outcomes were reported by Stolzenburg et al. (30) and Carbonara et al. (29). It was worth noting that the PSM rate obtained following RARP for large prostates in our analysis was somewhat lower than that (19%) reported by Stolzenburg et al. (30), which may be partly attributed to various contributing factors related to the characteristics of larger prostates, such as greater lead time bias and decreased PCa density (8). Larger PVs are also correlated with lower risks of PSMs and extracapsular invasion, as well as favorable pathologic characteristics, all of which contribute to favorable oncologic outcomes (9, 31). Moreover, the distances between the risks of PSMs and occurrences of robust clinical events were relatively remote, greatly depending upon preoperative elements such as the preoperative PSA, advanced clinical stage, and higher Gleason scores (13, 24). Indeed, we found relatively low BCR rates in the RARP group (7.9%) at a median follow-up time of 36.8 months and in the LRP group (8.6%) at a median follow-up of 32.8 months, which was consistent with the low BCR rates (10.5%) following RP for PCa with a large PV (≥50 ml) at a median follow-up period of 36.1 months in the study reported by Mandel et al. (32). The similarity in the BCR-free survival rates after RARP and LRP corroborated the comparative capability in oncologic control following the radical removal of localized PCa with a large prostate.

Our analysis showed that, compared with LRP, RARP resulted in improvements in postoperative return to UC and EF for localized PCa with large prostate dimensions, which agrees with the results of previous RCTs (20, 30) comparing RARP and LRP for localized PCa. Several pathophysiological factors may account for the occurrence of post-prostatectomy incontinence (PPI). Apart from the preoperative variables encompassing age at the time of operation, preexisting lower urinary tract symptoms, higher BMI, and bladder dysfunction, the structural damage to anatomic supporting structures and neural elements during the RP process may play a crucial part in the development of PPI (13, 33). Given the comparability of all the preoperative variables between the two arms in our analysis, the significant differences in UC and EF recovery observed between the RARP and LRP groups in our study could be attributed to the benefits of robotic platforms in preserving membranous urethral and nerve components and allowing the reconstruction of the surrounding supporting structures. Evidence regarding the impact of a large PV on functional outcomes following RP has yielded controversial results, thereby impeding the generalizability of conclusions (7, 9, 32). The UC rate detected at 12 months post-operation in the RARP group in our study was in line with the UC rate at 12 months after RARP in a published study by Porpiglia et al. (34). The superiority of robotic surgery in greater preservation of neurovascular components can be greatly responsible for the preferable EF recovery following RARP compared with LRP.

Some limitations must be taken into account when interpreting our findings. The retrospectively designed settings caused the structural drawbacks in collecting the included information. The study population, although well balanced between the two groups, was relatively small due to the strict limitations of the inclusion criteria. Certain complications, especially the ones grade II or lower, may be underestimated despite the meticulous application of all methods, including medical records, outpatient follow-up, and telephone interviews.

In spite of these shortcomings, however, to date, the present analysis is the first one concentrating on evaluating the perioperative, functional, and oncologic results of RARP and LRP for localized PCa with a large prostate (≥50 ml), which is of clinical significance. Our conclusions were drawn on the basis of outcomes analyzed over a minimum of 2 years follow-up and further strengthened in the foundations of the statistical comparability of all perioperative features between the two groups and the rigorous methodology applied.

## Conclusions

For the surgical management of localized PCa with a large prostate (≥50 ml), RARP had a tendency toward a lower risk of postoperative complications and better functional preservation without compromising cancer control when compared with LRP. Further prospective randomized studies with a larger sample size and sufficiently long follow-up periods are necessary to confirm our results.

## Data Availability Statement

The raw data supporting the conclusions of this article will be made available by the authors, without undue reservation.

## Author Contributions

BF and GW conceptualized and designed the study. WD and XL acquired the data. XL, LC, and CZ analyzed and interpreted the data. WD performed the statistical analysis. WD and WL wrote the manuscript. BF, GW, XZ, and JG edited the manuscript. All authors contributed to the article and approved the submitted version.

## Conflict of Interest

The authors declare that the research was conducted in the absence of any commercial or financial relationships that could be construed as a potential conflict of interest.

## Publisher’s Note

All claims expressed in this article are solely those of the authors and do not necessarily represent those of their affiliated organizations, or those of the publisher, the editors and the reviewers. Any product that may be evaluated in this article, or claim that may be made by its manufacturer, is not guaranteed or endorsed by the publisher.
